# Differently different?: A commentary on the emerging social cognitive neuroscience of female autism

**DOI:** 10.1186/s13293-024-00621-3

**Published:** 2024-06-13

**Authors:** Gina Rippon

**Affiliations:** Emeritus of Cognitive NeuroImaging, Institute of Health and Neurodevelopment, College of Health and Life Sciences, Aston University, Birmingham, B4 7ET UK

**Keywords:** Autism, Sex/gender, Social brain, fMRI, Male bias, Camouflaging

## Abstract

Autism is a neurodevelopmental condition, behaviourally identified, which is generally characterised by social communication differences, and restrictive and repetitive patterns of behaviour and interests. It has long been claimed that it is more common in males. This observed preponderance of males in autistic populations has served as a focussing framework in all spheres of autism-related issues, from recognition and diagnosis through to theoretical models and research agendas. One related issue is the near total absence of females in key research areas. For example, this paper reports a review of over 120 brain-imaging studies of social brain processes in autism that reveals that nearly 70% only included male participants or minimal numbers (just one or two) of females. Authors of such studies very rarely report that their cohorts are virtually female-free and discuss their findings as though applicable to all autistic individuals. The absence of females can be linked to exclusionary consequences of autism diagnostic procedures, which have mainly been developed on male-only cohorts. There is clear evidence that disproportionately large numbers of females do not meet diagnostic criteria and are then excluded from ongoing autism research. Another issue is a long-standing assumption that the female autism phenotype is broadly equivalent to that of the male autism phenotype. Thus, models derived from male-based studies could be applicable to females. However, it is now emerging that certain patterns of social behaviour may be very different in females. This includes a specific type of social behaviour called camouflaging or masking, linked to attempts to disguise autistic characteristics. With respect to research in the field of sex/gender cognitive neuroscience, there is emerging evidence of female differences in patterns of connectivity and/or activation in the social brain that are at odds with those reported in previous, male-only studies. Decades of research have excluded or overlooked females on the autistic spectrum, resulting in the construction of inaccurate and misleading cognitive neuroscience models, and missed opportunities to explore the brain bases of this highly complex condition. A note of warning needs to be sounded about inferences drawn from past research, but if future research addresses this problem of male bias, then a deeper understanding of autism as a whole, as well as in previously overlooked females, will start to emerge.

## Plain Language Summary

Autism is a neurodevelopmental condition, behaviourally identified, which is generally characterised by social communication differences, and restrictive and repetitive patterns of behaviour and interests. It has long been claimed that it is more common in males, with oft-quoted ratios of 4M: 1F. This has been reflected in the development of diagnostic criteria for autism and, consequently, of measures of eligibility for autism research programmes, with females being (as is now emerging) disproportionately excluded.

As outlined in this review, this issue has been particularly problematic in brain-based studies of autism. Many studies have only tested male autistic participants, or minimal numbers of autistic females. By default, sex differences were not examined. But the impression given by such research reports has commonly been that the findings would be applicable to all autistic individuals.

Recent psychological and clinical research has shown that there are a significant number of autistic females who have been missed by traditional diagnostic practices. Their inclusion has increased their eligibility for autism research studies. With respect to brain research, it has become possible to devise studies with matched numbers of autistic females and males, and to replicate studies that have previously only tested males. Newly emerging findings from such studies are demonstrating that the ‘robust’ autism-related differences previously observed in autistic male-only cohorts do not fully generalise to autistic females.

It will be necessary to exercise caution in drawing inferences from previous male-biased studies of the autistic brain. However, the identification and inclusion of previously excluded female autistic participants hopefully offers more accurate insights into this highly complex and heterogeneous condition.

## Highlights

- Several decades of neuroimaging research into autism has been based almost entirely on males; even big data sets show strong evidence of male bias.
- There is clear evidence that autistic females are being excluded from the research process by failures in diagnostic practices that have been developed on male-biased cohorts.
- Models of autism which inform research protocols are based on male autism phenotypes; it appears to have been assumed that the substantially fewer females that are diagnosed will present with equivalent, if milder, patterns of autistic differences.
- Newly emerging findings from social cognitive neuroscience research studies investigating sex/gender differences in autistic behaviour and associated biological correlates are demonstrating that the ‘robust’ autism-related differences previously observed in autistic male-only cohorts do not fully generalise to autistic females.
- Autism research programmes should prioritise the exploration of sex/gender effects.

## Introduction

Autism Spectrum Disorder or Condition (ASD or ASC—henceforth ‘autism’) is a lifelong neurodevelopmental condition, behaviourally identified, which is generally characterised by impairments in social and communicative skills and restrictive and repetitive patterns of behaviour and interests. It is usually identified within the first three to four years of life. Symptom profiles are very heterogeneous, presenting with a wide spectrum of difficulties and several levels of severity [1, 2]. Autism is estimated to affect 1% of children worldwide [3]. There has been an exponential increase in reported prevalence rates over the years [4]; the most recent statistics in the US quote rates of 1 in 36 children as being diagnosed as having autism [5] and a recent survey reported a 787% increase between 1998 and 2018 [6]. It is generally accepted that such increases reflect increase in public awareness and more proactive diagnostic practices.

### Functional brain differences in autism

Autism is a highly heritable condition, with estimated heritability ranging from 40 to 90%*,* and the search for the genetic bases and associated neural and behavioural correlates has been ongoing since 1970s [7–9]. As autism is clearly a brain-based condition, but only recognisable behaviourally, neuroimaging techniques, particularly those associated with social cognitive neuroscience, have proved fruitful, with over three thousand studies published since 1999. Initial localisationist studies focused on regionally specific structural comparisons, were mainly characterised by small, highly heterogeneous cohorts, resulting in inconsistent findings and lack of replication [10]. Subsequently, the focus shifted to structural and functional connectivity and/or identifying activation of task-related networks [11, 12]. Overall, such studies generally reported patterns of both structural and functional atypical connectivity in autism, but the findings have been mixed and at times contradictory [13, 14].

### Autism and the social brain—accumulating evidence

With social behaviour difficulties a key feature of autism, the emergence of the social brain hypothesis provided a profitable framework for autism research. A core premise of the social brain theory is that the human brain has evolved to be uniquely attuned to social interactions, well equipped to navigate the complexities of human society. Human brains are thus equipped with networks dedicated not only to self-referential processing but also to the processing of the thoughts, beliefs and intentions of others. Higher level social processing underpins the acquisition and retention of social knowledge and, in interaction with key emotional and motivational coding processes, enables the production and regulation of socially appropriate behaviour. Thus, it has been possible to operationalise the core processes of social behaviour and linked them to key neuronal structures and networks [15–17]. See Table 1.

**Table 1:** Social cognition processes and associated neural correlates

Social cognition processes	Neural correlates of social cognition
Social knowledge [17–20] • Sense of self (inc. self-monitoring) • Sense of others (inc. mentalising, person perception) • Social norms/social context • Social categories (e.g. ingroup/outgroup) • Social cues (e.g. eye gaze, face processing, body language, social prediction)	• *Default Mode Network* (DMN- inc. dorsomedial prefrontal cortex, posterior cingulate cortex, precuneus) • *Mentalising Network (*inc. Ventromedial prefrontal cortex, orbitofrontal cortex, Superior temporal Sulcus (STS); Temporal Parietal Junction (TPJ), Posterior Cingulate Cortex*)* • *Anterior Temporal Lobes* ( anterior medial temporal cortex)
Social motivation [20–22] • Prosocial behaviour (e.g. empathy, co-operation) • Salience detection • Belongingness/social exclusion/social pain • Social reward/punishment processing • Emotional feedback	• *Salience network* (inc. ventrolateral prefrontal cortex (vlPFC), dorsolateral prefrontal cortex (dlPFC), anterior insula, ACC) • Orbitofrontal cortex • Striatum (caudate nucleus, putamen, nucleus accumbens) • Amygdala
Social regulation [23–26] • Behavioural choice (error evaluation/conflict monitoring) • Social attention • Response inhibition/Impulse control • Emotional regulation	• Ventrolateral prefrontal cortex • Dorsolateral prefrontal cortex • ACC • Amygdala • Insula

With respect to autism, this framework could then be applied to those areas of social behaviour which had been identified as atypical, such as difficulties with emotional recognition or low levels of social engagement, and thereby target potential structural and functional regions of interest in the autistic brain. This offered a promising way forward for a deeper understanding of the neural bases of autism.

As the number of studies into the social neuroscience of autism accumulated, it became possible to carry out reviews and meta-analyses, including coordinate-based activation likelihood (ALE) estimations of neuroimaging data [27]*.* One of the first such summaries was an ALE meta-analysis of functional brain correlates of social and non-social processes in autism. A review of 39 functional imaging studies (37 using fMRI, 2 using PET), implicated consistent patterns of hypoactivation in the anterior cingulate and right anterior insula in social tasks [28].

In 2016, another ALE review examined 50 different fMRI studies of social cognition, defined as tasks focussing on social information processing tasks, such as judging facial emotional expression [29]. Group differences indicated underactivity in autistic participants in several social brain areas, such as the amygdala, superior temporal gyrus and the cingulate cortex.

In 2018, a systematic review and meta-analysis of 13 brain imaging studies linked to the social motivation hypothesis, that autistic individuals find social stimuli less rewarding than neurotypicals [30], revealed large clusters of reward circuitry hypoactivation in the autism group in bilateral caudate and ACC regions [31]. An additional systematic review of research into the social motivation hypothesis reported on 27 papers, including 8 fMRI studies [32]. A summary of these studies indicated hypo-activity in key parts of the social brain and associated regions such as the salience network, amygdala, nucleus accumbens, caudate nucleus, ACC, insula and ventral striatum*.*

A narrative review of the default mode network and its association with social cognition reported on 29 studies of DMN (resting state) connectivity in autistic adolescents [33]. 15 of the 29 studies found predominant underconnectivity in the DMN, associated with social impairments.

And finally, an ALE review summarised fMRI data from 23 different studies of the functional architecture of the reward processing system in autism, involving a range of different reward paradigms, both social and non-social. The authors concluded there was robust evidence for an underactive striatal system in autistic individuals, evident mainly in the right putamen and right nucleus accumbens. This was equated behaviourally with:”…diminished pleasure, reduced motivation to acquire regarding stimuli and less avoidance of punishment.”[34].

Developmental aspects of the functional correlates of social behaviour in autism were also being explored, with the outcomes of child studies being compared with those from adults, in order to investigate age-related changes. An ALE meta-analysis of 18 child studies and 24 adult studies revealed age-related differences in patterns of both hyper- and hypo-activation in fronto-temporal structures, identifying the need for longitudinal approaches to research in this area [35].

So, between the years of 1990 and 2020 (with most studies in the last decade), accumulating neuroimaging evidence from well over 100 studies was generating a model of a characteristically under-active reward system in autism, linked to key nodes of the social brain, particularly the striatum. This resonated well with the classic autism phenotype of a socially withdrawn individual, apparently lacking in the motivation to form affective contact with others [36]. There were even suggestions that the striatum might be a promising target for neuromodulation [34].

But it emerges that this promising development was based on incomplete data. Closer examination of the cohorts in each of the studies in each of these meta-analyses revealed that, in many cases, only male autistic participants were tested—in many of the remainder, only one or possibly two females could be found. 45% of the studies only included autistic males, with a further 24% adding just 1 or 2 females. Of the more than four thousand autistic participants tested overall, less than 10% were female. Looking at each survey and averaging across the cohorts from each of the studies reported, ASD M: F ratios ranged from 27:1 to 6.29:1; put another way, the percentage of females tested ranged from 3.5% to 13.7%. See Table 2.

**Table 2:** Reviews of studies into social brain connectivity/activation in autism

Review authors (time range of studies reviewed)	N of studies in review (duplicates removed)	Patterns of activation/Connectivity In ASD (brain areas emphasised)	N of Male only Studies (+ 1 or 2 F)	Total ASD participants M/F
Di Martino et al., 2009 (1990–2008)	24	Hypoactivation (esp. ACC and insula)	17 (7)	270/10
Dickstein et al., 2013 (1999–2011)	10	Hypoactivation (in ASD adults)	6 (1)	124/11
Patriquin et al., 2016 (1992–2014)	30	Hypoactivation (STS,TPJ,amygdala,insula)	14 (11)	368/44
Clements et al., 2018 (2010–2018)	12	Hypoactivation (ACC, bilateral caudate)	6 (2)	219/24
Bottini, 2018 (2010–2015)	6	Hypoactivation (dorsal striatum, left caudate)	2 (2)	98/10
Nair et al., 2020 (2010–2019)	29	Hypoconnectivity (DMN)	8 (3)	2397/263
Janouschek et al., 2021 2007–2020)	13	Hypoactivation (striatum)	4 (4)	239/38

Obviously none of the 124 studies in the above reviews carried out any sex different analyses; three of the seven reviews did note this lack. And the studies identified in the reviews almost invariably reported their findings in terms of ‘autism’ or ‘ASD’ or ‘ASD youth’, for example, not drawing attention to the fact that they were only or mostly studying males. Although a M/F difference in the prevalence of autism has, as we shall see, long been asserted as a possibly defining characteristic of the condition, there *are* females on the spectrum and the near absence of their representation in these and other such studies should be a cause for concern. In a recent survey of twenty years of sex/gender autism brain imaging research, of the 1428 articles on brain structure and function in autism, 30% only studied male participants (and 0.28% studied only females); 77% of the remaining studies were rejected because sex/gender variables were not assessed or just treated as a covariate in analyses [37].

### The male spotlight problem in autism

Almost since the emergence of the initial descriptions of autism, one oft-repeated statistic is its greater prevalence in males. The reported male: female ratio has sometimes been stated as high as 15:1 [38] but, until recently, the most commonly accepted estimate was 4:1[39].However, a more recent, systematically-calculated estimate of autism prevalence studies indicated a ratio of below 3.5:1. [40].

The observed preponderance of males in autistic populations has served as a long-standing framework in all spheres of autism-related issues, from recognition and diagnosis, through to public awareness and stereotypical media representation, to theoretical models as well as cutting-edge research agendas. It may also have created a series of obstacles in the sphere of sex differences research in autism [41].

Initially, there is the problem of ascertainment bias, where some members of a target population are excluded. With respect to autism, there is increasing evidence that current diagnostic practices, including referral and testing, are biased against females. Clinician awareness of the autism-as-male model has resulted in both qualitative and quantitative reports of refusals and/or delays in referrals of girls for testing [42, 43]. Scrutiny of the ‘gold standard’ tests for autism diagnosis, the Autism Diagnostic Schedule (ADOS-2) and the Autism Diagnostic Interview Revised (ADI-R) [44, 45] has revealed problematic male biases in the formulation of such tests, including non-gendered norms for cut-off points and the male-focussed nature of the associated observational scenarios and interview schedules devised for assessing atypical patterns of behaviour [46–49]. The Loomes et al. study mentioned above that reported a lower M:F ratio than that traditionally accepted, noted that this was mostly evident in studies using active ascertainment methods by community-based sampling of autism traits rather than passive acceptance of pre-existing autism diagnoses. A recent statistical modelling approach to measuring the male bias problem in both recognition and diagnoses suggests that, with current diagnostic practices, as many as 80% of autistic females could suffer from refused or delayed assessment, or misdiagnosis into other categories such as borderline personality disorder [50]. So, with respect to potential research cohorts, the pool of female participants may be severely restricted by the ‘gatekeeping’ effect of commonly used tests..

An additional source of data loss is the so-called ‘leaky recruitment-to-research pipeline’ problem. The use of the above-mentioned gold standard tests has commonly been a requirement for autism research funding. So even where recruitment and screening is community-based, and a potential pool of participants have been identified, for example by the use of autistic trait questionnaires [51, 52] researchers may then be required to use ADOS and/or ADI tests to select their final cohorts. A recent study from a group at MIT demonstrated that this can result in the exclusion of females at a rate over 2.5 times higher than males [53]. ADOS assessment tests were retrospectively applied to 145 adults whose autism had been established via a community diagnosis in order to participate in ongoing research. After ADOS administration, 25 of the community-diagnosed females (50% of the original cohort) were excluded from further research participation, compared with only 19% of the males. This shifted the male: female ratio from 1.9:1 to 3.1:1 in the final research sample.

### Under-studied females—the use of big data sets

There is universal agreement that autism, as currently defined, is a hugely heterogeneous condition, characterised by the well-known saying “….if you’ve met one person with autism, you’ve met one person with autism”. A wide range of symptom profiles and levels of severity are encompassed within autism spectrum measures, which then feeds into a variety of genetic explanations and biological models. This heterogeneity impacts on any scientific study of autism, particularly where population-level comparisons are made, as such variability can mask potentially relevant differences.

Increasing the size of available samples and/or data by combining information from international, multidisciplinary centres was proposed as a way of increasing the scale of autism studies, as well as improving issues of cross- laboratory robustness and reproducibility of findings [54]. Acknowledgment of the advantages of this approach plus associated funding opportunities has led to the establishment of several big data sets. A review in 2017 reported on 33 such resources [55], ranging from 50,000 cases in the SPARK data base [56] to 42 fMRI brain connectivity matrices then available in the Human Connectome project [57]. Additional such resources include the EU-AIMS Longitudinal European Autism Project (LEAP) [58] and the MRC Autism Imaging Multicentre Study (AIMS) [59].

Big data sets could address biological sex as a source of heterogeneity, even where there is an accepted imbalance in male–female prevalence data. Limitations on studying ‘rarer’ females on the spectrum could be overcome, at least partly, where wide-scale recruitment and/or combination of multi-site data sets results in a sufficiently large pool of female autistic participants to enable meaningful group comparisons [60]. However, the initial use of big data sets did not appear concerned with sex/gender issues in autism. For example, the ‘inaugural’ paper from the first iteration of the Autism Brain Imaging Data Exchange (ABIDE-1), launched in 2012 and containing over a thousand fMRI resting-state datasets and phenotypic data sets from over 539 diagnosed autistic individuals, only reported on analyses of male autistic participants (N = 360). Scrutiny of the ABIDE 1 dataset reveals that 485 (88%) of the data sets were from males; 25% of the sites invited to participate had anyway excluded females “by design” [61].

A second iteration was launched in 2016, adding another 487 autistic datasets, increasing the number of females from 65 to 138, at this point specifically drawing attention to sex-related differences as a possible source of heterogeneity [62]. The female data set still only comprised 15% of the total, which was deemed a reflection of the higher prevalence in males (although not a reflection of even the traditional 4:1 ratio).

One aspect of these autism data sets is that eligibility for inclusion almost invariably involves confirmation via the use of the gold standard ADOS and/or ADI tests. The MIT group that had revealed the consequences for their own data set of this practice, also surveyed several large publicly available autism data sets (SPARK, ABIDE II and I). They found that where ADOS had been used to determine inclusion, the male: female ratio was of the order of 7:1. Where the data sets used community diagnoses, the ratios varied from 0.68 to 1.8:1.

An additional feature of this ascertainment problem is that, as it has been demonstrated that there is a male bias in the test materials and the clinical thresholds, those females who do acquire an autism diagnosis may, de facto, be more similar to males on the spectrum. Where female-male comparisons are part of a study rationale, the study design should reflect appropriate sample sizes to ensure adequate statistical power [63].

In the case of sex-difference autism studies, given the possibility that differences may be minimised by selection factors (as in the use of ADOS), sample sizes should be as balanced as possible. As shown above, even recruiting to the alleged 4:1 male: female ratio is rare and would anyway not ensure sufficient statistical power. This should be borne in mind when assessing autism sex-difference studies using big data bases that did not find those sex differences which might have been predicted from preceding small-scale studies. Acknowledging that such studies may themselves be statistically inadequate [64], they should not be too readily dismissed on the basis of large-scale studies which themselves have not overcome all analytical hurdles.

For example, Ypma et al. [65], reported DMN hypo-connectivity in both male and female autistic groups drawn from ABIDE I, based on a comparison of 408 males and 55 females. They noted that their findings demonstrated that hypo-connectivity, previously shown to characterise males on the spectrum was also “robustly present” in females.

Similarly, Moessnang et al. [66] 58 reported on a task-based fMRI study of 151 autistic males and 51 autistic females (with 123/66 male/female controls) drawn from the Longitudinal European Autism Project – LEAP, and failed to find effects of either sex or diagnosis.

Ilioska et al. [67], drawing on data from LEAP and both ABIDE I and II, investigated patterns of hyper- and hypo- connectivity in 655 autistic males and 141 autistic females (with 772/256 male/female controls) [67].Patterns of both hyper- and hypo-connectivity were associated with social impairments. No sex differences were shown, although tested for. The authors did comment on the need for replication in samples with a more balanced male-to-female ratio.

Studies based on well-defined inclusion criteria for the selection and matching of participants have yielded more promising insights. For example, Alaerts et al. [68] selected 42 matched male and female autistic cases from the ABIDE I dataset and compared patterns of resting-state functional connectivity. Autistic males showed a highly consistent pattern of whole-brain hypo-connectivity, whereas females showed an overall pattern of hyper-connectivity. This stands in some contrast to the findings from the studies outlined above, drawing on the same dataset, but with a significantly unbalanced cohort.

Big data sets are obviously an enormously valuable resource in autism research but, with respect to studying the role of sex-based differences in all aspects of this condition, may have imported some of the ‘male spotlight’ difficulties, both in their construction and their use, which could have negatively impacted on research progress in this area. This should be borne in mind, both in the use of such databases, but also in drawing on their research outputs to design studies, generate hypotheses and interpret findings. In addition, where a data-driven, ‘discovery science’ approach is taken in interrogating such data sets [69], researchers should be alert to potential confounds linked to recruitment protocols, and may wish to consider more careful profiling and matching of their autistic participants based on narrow rather than broad constructs [70, 71].

### What happens when you do include females?

In 2017, as part of the US Autism Centre of Excellence network, a multi-disciplinary research consortium specifically designed to address issues of sex and gender in autism research was launched. Gender Explorations of Neurogenetics and Development to Advance Autism Research (GENDAAR) combines multi-site genetic, neuroimaging and phenotypical data from well-matched samples of girls and boys with and without autism. Findings from this program are adding to the emerging body of evidence that ‘robust’ findings in male autistic participants do not always generalise to females. This is demonstrated by two studies on social reward processing the authors of which which include members of the GENDAAR consortium.

The first study was carried out on sixteen autistic males and reported diminished neural response to social rewards, particularly in the ventral striatum [72].This had been interpreted as consistent with the autism phenotype of reduced social motivation. The second study recruited 39 females and 43 males from the GENDAAR cohorts [73]. They found that autistic girls displayed *increased* activity to social rewards, especially in the nucleus accumbens, compared to autistic boys. The autistic girls also showed greater reactivity in the anterior insula compared to typically developing girls. These patterns of neural activity would be consistent with *higher* levels of social motivation, i.e. inconsistent with the accepted autism phenotype (and with previous neuroimaging findings based only on males).

In similar vein, a study in 2016 on sensory over-responsivity in autism, investigated resting-state connectivity in the salience network [74]. (Atypical sensory responsivity was added as a core characteristic of autism in the latest iteration of DSM; there are consistent reports that it is more common in autistic females [75, 76]). The findings reported increased resting-state functional connectivity between salience network nodes (such as the anterior insula and the amygdala) and primary sensory processing areas in the brain. This correlated with behavioural measures of sensory over-responsivity. The effect was less evident in visual association areas, which was interpreted as evidence of decreased attention to social information, consistent with the traditional autism phenotype. The autistic cohort in this study comprised 27 males and 1 female.

In 2020, several members of this group again investigated the relationship between salience network connectivity and measures of sensory over-responsivity, this time with 16 females and 37 males in the autistic cohort [77]. As in the previous study, males showed strong functional connectivity between the salience and the primary sensory networks, strongly associated with sensory over-responsivity. For females, however, sensory over-responsivity was more strongly associated with increased functional connectivity between the salience network and the pre-frontal regions, including the anterior cingulate. This was speculatively linked to a female autistic tendency to regulate emotional consequences to sensory over-responsivity and avoid social embarrassment.

A study of resting-state functional connectivity in the mentalising system was based on equal numbers of matched female and male autistic participants (N = 48), selected from the ABIDE 1 data set [78]. Females showed hypoconnectivity between the medial prefrontal cortex, the precuneus and the right TPJ; males showed hyper-connectivity of the bilateral TPJ. An interaction between sex and autism was found in both short- and long-distance functional connectivity, generally with autistic females showing underconnectivity and ASD males showing overconnectivity.

These findings are partly consistent with a study in 2016 on patterns of activity in the mentalising network, a key neural substrate of the ‘mindreading’ aspect of social behaviour [79]. The cohort was small, but there were near equal number of female and male participants (14 female, 13 male).Whole brain analysis revealed decreased activity in the superior temporal sulcus in autistic males compared to control males while processing social information; no activation differences were found between autistic and control females.

In 2017, a review from the GENDAAR consortium summarised 67 different studies of resting state measures in autism, in order to profile connectivity findings [80]. 18 of these studies excluded females altogether, and there was a M: F ratio of 8.33:1 in the overall total of autistic participants (10,725 M/ 1287 F). 13 of the studies included a focus on the DMN, with the majority reporting under-connectivity. 6 of these studies had no female autistic participants, and females only represented 9.3% of the overall total. For example, a male-only (N = 25) study by von dem Hagen et al. [81] reported patterns of reduced functional connectivity between and within such social networks, interpreted as causing impaired processing of social signals in autism, because of difficulties in communication and integration across the networks. The authors of the review specifically commented on the dearth of females, but the overall profiling of patterns of connectivity has regularly been cited, included DMN under-connectivity.

A partial update in 2020, again from the GENDAAR consortium, reported on functional connectivity in key social brain networks, the Default Mode Network and Central Executive Networks, as well as the salience network [82]. The study was based on comparison between carefully matched females (N = 34) and males (N = 46). Findings included evidence of greater functional connectivity in autistic girls between the DMN and the CEN than in their male counterparts.

Thus a picture emerges, that more recent brain imaging studies which include near equal numbers of females and males, indicate that a revision is required of earlier male-based neuroscience studies of autism. These have commonly reported apparently consistent findings of, for example, low levels of resting-state connectivity in key areas of the brain, particularly those associated with social behaviour and reward processing, and have linked these to a traditional (male-based) phenotype of limited social engagement and/or atypical sensory responsivity. It is also worth noting that those studies that have found sex differences are consistent with an observation made in the 20 year survey of sex/gender differences in human autistic brains mentioned above, that studies with low male–female participant ratios were much more likely to report positive findings. However, it should also be noted that, currently, studies with near equal male: female numbers are also characterised by small sample sizes, with the accompanying danger of false positives. This indicates a clear need of replication studies drawing on larger samples where problems with ascertainment bias have been overcome, and the increased number of female participants affords more representative sampling opportunities.

A significant footnote to this emerging story of the consequences of a focus on sex differences in autism has emerged from the GENDAAR consortium. The findings are linked to research into the Female Protective Effect, a model proposing that female biology provides some kind of buffer against the expression of autism-linked genetic factors, which is taken to account for the lower incidence of autism in females. Evidence showing that autistic females may show greater prevalence of candidate mutations has supported this concept [83].

In addition, this approach offers the possibility of linking autism genotypes with neuroimaging and behavioural phenotypes, with exploration of sex differences at the heart of such research. A study in 2020 investigated the relationship between variants of the receptor gene for oxytocin (OXTR), a hormone linked to social behaviour, and resting-state functional connectivity in key hubs of the brain’s reward network, in females and males with and without autism [84]. In autistic females (N = 50), there was a positive relationship between the OXTR risk-allele load and increased connectivity between the nucleus accumbens, subcortical regions and prefrontal areas involved in mentalising, as compared to autistic males. In autistic males (N = 37), a higher OXTR risk-allele load was associated with reduced within-network connectivity.

A study in 2021 measured fMRI responses to a social motion task in 94 autistic participants (46 female, 48 males) and 113 controls (54 females, 59 males) [85]. A key finding was that autistic females showed lower level of activation than control females in parts of the striatum. This might seem at odds with reports of higher levels of striatal activation in autistic females, but these were associated with social reward task as opposed to the social perception task used here. Consistent with the FPE model, autistic females showed a greater load of genetic abnormalities, significantly in genes commonly expressed in striatal development. This effect was not evident in males.

A third paper from the GENDAAR consortium focussed on connectivity in the salience network [86]. Their findings indicated that genome-wide risk for autism appeared to affect females and males differently. In autistic males (N = 30), elevated genetic risk was associated with increased connectivity between the salience network and somato-sensory processing regions, potentially linked to atypical types of repetitive behaviours. In autistic females (N = 31), despite increased genetic load, salience network connectivity was not affected, interpreted as evidence of some kind of protective factor preserving striatal function from the impact of genetic risk, and preventing diagnostically significant repetitive behaviours.

Clearly, the output from such studies are at a relatively early stage. And the studies themselves, although involving near-equal numbers of females and males, are relatively small-scale, thereby potentially open to false positive problems and in need of replication. But they add to an emerging body of evidence of multi-level sex x diagnosis interactions in autistic brains which would obviously not have been revealed even as little as five years ago, when seemingly revelatory findings in the neuroscience of autism were based on male-only studies.

### Missed or missing? Is ‘female’ autism different?

An additional aspect of understanding the male bias issue in autism is that females are missing from the autism statistics, not just because the diagnostic schedules are poor at spotting them, but because ‘female’ autism presents differently, with a different symptom profile and patterns of behaviour. Until the 1980s, there was surprisingly little attention paid to the possibility that autism might present differently in females and that this might be part of the apparent sex bias. Phenotyping of autism in girls was effectively based on taking the male autistic profile as given and assessing the degree to which the female autistic profile matched this. So generalised, ‘less than’, summaries, reported that, on average, females tended to score lower on measures of restricted interests and repetitive behaviours, and lower on measures of social dysfunction [70, 87, 88]. This ‘milder’ presentation, when assessed by diagnostic schedules based on the male image of autism, resulted in many females failing to reach the clinical threshold, thus sustaining the self-fulfilling impression of autism as primarily a male condition.

However, one espoused view is that females ‘hovered’ below clinical thresholds not necessarily because their symptoms were milder, but because they were better at disguising them. There had, indeed, been an early suggestion that girls might be better at disguising or camouflaging their autistic symptoms thereby ‘flying beneath the diagnostic radar’ [38, 89].

Linked to this explanation was a social constructivist perspective, highlighting the role of gendered socialisation. An emphasis on social compliance in girls thereby rendered them better able to employ relevant social skills and minimise the social impairments characteristic of autism [90, 91]. So the inference was that autism in girls *was* like autism in boys, but the girls were better at hiding it.

However, in the last decade or so, emerging evidence suggests that autism in girls may not just be a milder version of autism in boys, but that core aspects of their autism are markedly different (and thus, indeed, less likely to be picked up by diagnostic instruments designed around male cohorts). A key difference appears to be a much more powerful drive for social engagement and belongingness [92, 93]. And entangled with this aspect of their autism appears to be the need to disguise their autistic differences and difficulties, by adopting types of behaviour variously referred to as camouflaging or masking. Research indicates that this is both more extreme (and maladaptive) than gendered social compliance [93–98].

A wave of powerful personal testimonies from autistic females who had been diagnosed in adulthood suggested that, not only were genuninely autistic women missing from autism statistics because the current diagnostic practices had failed to recognise them, or because of powerful biases based on the belief of autism as predominantly a male problem, but because of a particular set of behaviours which served to, superficially, counteract or mask classic symptoms of autism [99–105].

Qualitative and quantitative analyses of such reports of late diagnoses in women, revealed a characteristic lifelong pattern of ‘social coping’ in such cases. This has variously been described as camouflaging or masking or adaptive morphing. It comprises various strategies adopted by some autistic individuals, usually female, to disguise or compensate for autism-related difficulties and differences. This may involve intensive study and mimicking of the social behaviour of others, particularly peer groups, with the specific aim to blend in and not be noticed as different. It can involve the conscious generation of a form of social script—how to make small talk, how to maintain eye contact, how to laugh at jokes—which will be rehearsed and followed whenever a social situation is encountered [106–115].

Camouflaging has become a key focus in recent clinical and psychological research into autism [116, 117]. It has been operationalised as a discrepancy between standard internal measures of autistic traits and external presentation of social behaviour, such as emotion recognition. A Camouflaging Autistic Traits Questionnaire has also been developed [116, 118–120]. Emerging data show that it is much more common in autistic females or females with high levels of autistic traits [112].

If camouflaging is the product of a need to disguise autistic differences, which has succeeded to the extent that autistic females are diagnosed later, if at all, it might be therefore be viewed as a successful ‘survival strategy’ [114]. Unfortunately, there is clear evidence that it can also be a maladaptive and damaging pattern of behaviour. It is associated with reports of high levels of anxiety, exhaustion and stress, as well as suicidal ideation and chronic depression. This is evidenced not only by self-report but by proof of lifelong struggles with mental health alongside similarly lifelong signs of camouflaging behaviour [95–98, 121].

### Camouflaging and the social brain

Camouflaging encapsulates key aspects of social behaviour. The close attention to social cues and the production and rehearsal of social scripts indicates high-level engagement with the acquisition and processing of social knowledge [122] Additionally, camouflaging as a coping strategy embodies many of the regulatory aspects of interactive social behaviour, including action selection, such as mimicking gestures or purposefully maintaining eye contact, or consciously suppressing autistic-like behaviours like stimming [123].

Self-report and interview outcomes emphasises the ‘impression management’ aspect of camouflaging behaviour as a ‘survival mechanism’, changing ways of responding in order to minimise evidence of difference and to maximise inclusion. Both qualitative and quantitative data indicate that a desire to fit in, to avoid the stigma of autism, is the main driving force behind camouflaging behaviour [94, 107, 124–126]. This resonates with the role of social motivation, belongingness and the fear of rejection or ostracism. Perhaps more than anything, the persistence of camouflaging behaviour, despite its association with high levels of mental health problems in autistic individuals, especially females, further indicates a powerful motivational force behind such conscious or unconscious social decisions which are superficially effective, but ultimately maladaptive [97, 109].

Camouflaging in autism, therefore, could prove to be a useful index of atypical social processing at the level of both brain and behaviour. Particularly given the accumulating (if still biased) evidence of abnormalities in the social reward circuits in the autistic brain, this could be a fruitful focus for research, with levels of camouflaging as an independent variable.

### Sex-differences in camouflaging: social brain networks/reward circuits as candidate pathways?

A study by Lai and colleagues from the MRC AIMS consortium investigated patterns of activation in the right TPJ and vmPFC components of the mentalising network during a classic scanner-based self-reference task [128]. At the behavioural level, there was no differences between autistic participants and controls on the self-/other-reference task, apart from faster RTs in females. With respect to neural processes, lower levels of activation in the vmPFC and the TPJ were evident in autistic males as compared to typically developing males; there were no differences between autistic and typically developing females. As above, reduced activation in social brain areas in autistic males is consistent with many previous studies. Camouflaging behaviour was measured as the discrepancy between intrinsic, self-rated autistic traits and external measures of atypical behaviour such as emotion recognition. Autistic women scored higher on this measure of camouflaging.

In autistic females, there was a significant positive correlation between vmPFC activation during the self-reference task and camouflaging scores, not found in autistic males. The authors speculated that the relationship between neural self-representation effects and camouflaging in females reflected a deeper form of camouflaging …” autistic women may engage substantial insight about their own behaviours in interpersonal and social contexts—specifically, how their behaviours impact others, gauging and managing the impressions they make on others, updating the differences between their natural and camouflaged behaviours, and how such behaviours will achieved the desired goal of being perceived as neurotypical” (p. 1219).

Walsh et al. [127] explored the relationship between patterns of brain connectivity in reward circuits, measured by fMRI, linked to patterns of compensatory, camouflaging behaviour. The autistic sample, 24F, 21 M, was selected to maximise detection of sex-related brain-behaviour associations. Camouflaging was measured by use of the Camouflaging Autistic Traits Questionnaire (CAT-Q) developed by Hull et al. [118].Higher levels of camouflaging in autistic females were associated with increased connectivity in reward pathways, including the right anterior cingulate, as well as in hypothalamic-limbic connections. In males, more positive functional connectivity in the anterior cingulate was linked to less camouflaging. This study also included measures of structural connectivity that were consistent with the sex differences in functional measures. The authors conclude that a focus on the relationship between camouflaging behaviour and patterns of connectivity and activation in reward pathways could offer a fruitful way forward in unpicking sex differences in the complex, multi-level associations between brain and behaviour in autism.

### General discussion and critical issues for future research

This review has shown that, once there is a specific focus on potential sex differences in the social cognitive neuroscience of autism, distinctive differences between the brains of autistic females and males can emerge. Recent reports of different patterns of connectivity and activation in the social reward system in autistic females as compared to males, associated with the apparently socially-driven camouflaging behaviour more commonly found in autistic females, are producing a markedly different picture from the traditional neuroscience models of autism, mainly developed using only male participants.

The evidence that autistic girls and women have been excluded from potentially valuable research programmes, either passively because of diagnostic practices or actively because of ascertainment bias in research eligibility criteria, is somewhat paradoxical. The male: female bias in autism is often quoted as one of the fundamental reasons for investigating sex differences in the brain, in order to provide a platform for autism research [129]. Yet the very arena that might offer valuable insights into sex/gender differences in autism has been characterised by a focus almost exclusively on males.

Traditional biological explanations of the apparently greater occurrence of autism in males have been couched in terms of, for example, some kind of hormonally-determined male vulnerability factor or of a genetically-related female protective effect that raises the threshold for clinical presentation [130]. This would seem to flag a clear agenda for a research focus on sex differences. Yet in many autism research fields, there seems to have been an assumption that, once females had ‘passed’ the diagnostic threshold, there was an equivalence of presentation with that of males. Default male models were applied to the development of diagnostic tools with, for example, no gendered norms to inform diagnostic algorithms. There was no separate characterisation of autistic behaviour as it presented in females. As we have seen, large numbers of brain imaging autism research studies did not measure sex differences, with the availability of female participants anyway being severely curtailed by many barriers, including those of recognition and referral, as well as active exclusion [41]. This was often not acknowledged in research reports, where findings were interpreted in generic terms, apparently referring to autism as a whole, with no attention drawn to the fact that the studies had been carried out almost exclusively on males.

There is a related example of the paradoxical nature of the dearth of females in autism research. In 2015, Lai et al. published a seminal paper, urging the autism research field to pay attention to sex and gender differences in autism, and setting an agenda for such research [131]. One issue they identified was that of sex or gender dependent characteristics—to what extent were differences in autistic brains and behaviour a function of typical differences in brain and behaviour? Of the findings discussed in this paper, for example, would it not be worth exploring whether the differences in social reward sensitivity in autistic females, evident at the level of both brain and behaviour, are a reflection of similar differences in all females? This is clearly a question that cannot be fully addressed when females are not included in relevant studies.

Additionally, as well as a lack of exploration of biological sex in autism neuroscience research, even less attention has been paid to the potential effects of gender, the role of external socialisation and experiential factors in both typical and atypical brain development. If, for example, there are female/male differences in social brain connectivity in the autistic brain, to what extent are these solely a function of sex-related brain characteristics or how much might they reflect continual exposure to gendered experiences, attitudes and expectations [132, 133]? And how much more might this be true of individuals perceived as atypical, such as autistic females, who may be exposed to higher levels of brain-changing experiences including bullying or abuse [134–136]. In addition, given evidence of the greater prevalence of gender variance in the autism community (refs), categorising autistic participants only as either female or male may be an equivalent disservice to assuming they are, by default, all male [137–140].

This raises the issue of a wider challenge to the existing binary female/male model which, to date, has mainly informed the full gamut of autism research, including brain differences. Different ways of exploring these are emerging with the advent of, for example, the notion of brains as unique mosaics of structural characteristics [141, 142]. Although incorporating previously overlooked autistic females into neuroscience research is certainly a step in the right direction, careful attention should be paid to the extent to which pre-existing assumptions about the binary nature of brains might distort research enquiries [143].

A wider context of the issues discussed here is the need to ask better questions in autism research, to avoid the problems outlined above. The use of participatory methods, the direct inclusion of autistic individuals in the research process via personal testimonies and detailed qualitative interview-based research data, has proved invaluable in gaining deeper insights into the condition. The understanding of camouflaging and its adaptation into autism’s research portfolio has been powerfully driven by the involvement of late-diagnosed autistic women. Almost by definition, the research community would not have understood the process of camouflaging and masking without listening to the lived experiences of autistic women [106]. Given that autism is a condition currently still identified by behavioural profiling, the quality of autism neuroscience research can be greatly enhanced by harnessing the insights offered by the autism community themselves [144].

## Limitations

A key focus of this review has been to draw attention to the dearth of neuroimaging-based research into female/male differences in autism, with until very recently many studies using male-only or male-biased cohorts. The commentary has emphasised this by concentrating, firstly, on the near complete absence of female participants in the majority of relevant research up until the last eight years or so, and then demonstrating the different patterns of results that have started to emerge once female participants have been included in effective numbers.

The omission of reference to other factors, such as intellectual disability, for example, is not to suggest their lesser relevance but is a result of attempts to manage, in the first instance, the complexity of the autism research literature. It is acknowledged that that the male bias in autism prevalence appears to vary as a function of intellectual ability [145] and that this should be factored into subsequent reviews of sex-related influences in autism.

Relatedly, there is no separate focus here on age and/or longitudinal studies. This could prove profitable for a second-stage review, as there is evidence that patterns of connectivity can change from childhood to adulthood [146]. There is also emerging evidence that sex-differences in brain and behaviour in autistic cohorts may be amplified during adolescence, so a focus on this age-related factor could be informative [147].

The presence of co-occurring mental health problems can be a confounding factor in sex-related autism research, as, on average, these are more common in autistic females [96] This was not primarily considered in this commentary, although the association between camouflaging behaviour and mental health problems has been acknowledged; such factors are often screened out in more fundamental neuroimaging research but, again, should figure in future assessments of ongoing investigations.

The focus of this review has been on research based on a traditional binary model of biological sex and its role in our understanding of autism, with discussions in terms of participants being categorised as either female or male. There has been no discussion of the role of variations in gender roles or gender identity as separate or entangled variables. This is another key omission in autism research to date, particularly in the light of evidence that gender nonconformity and gender diversity are significantly more common in the autism community [137–140]. This, in itself, could provide valuable insights into autism-related variations in social behaviour, including those related to gender identity and sense of self.

## Summary and conclusion

This review highlights a problematic practice within autism neuroscience research, of developing and testing brain-based models on male-only or heavily male-biased cohorts. For example, in a survey of over one hundred studies of the social brain in autism, 45% tested only male participants, with an additional 24% only including one or two females. This can be related to ascertainment bias in both the diagnosis of autism in females, and in the assessment of autistic female eligibility for inclusion in research. This has, until recently, also affected the construction of big data sets for data-sharing initiatives, thereby introducing male bias into many research programmes.

Consideration of more recent brain imaging research, with balanced cohorts of autistic female and male participants, indicates that the male bias in earlier studies has resulted in misleading characterisation of the neural correlates of key aspects of autistic differences, particularly with respect to social behaviour. Reports of different patterns of connectivity and activation in the social reward system in autistic females as compared to males, associated with the apparently socially-driven camouflaging behaviour more commonly found in autistic females, are producing a markedly different picture from the traditional neuroscience models of autism, mainly developed using only male participants.

This has also highlighted the under-recognition of behavioural differences in autistic females, specifically evidence of an enhanced social drive, indeed by camouflaging or masking behaviour, at odds with traditional profiles of autistic behaviour. As camouflaging is a pattern of autistic behaviour that, on average, is more common in females than males, targeting social reward circuits in the context of camouflaging behaviour could advance the understanding of sex/gender differences in autism [127, 128].

The findings of this review indicate the need for caution with respect to the generalisability of past autism research findings. There is a need for greater transparency with respect to highlighting the female/male breakdown in cohort demographics, and greater clarity in the discussion and interpretation of data that have been collected from male-only or male-biased cohorts.

The historical absence of females from many different clinical and empirical autism research agendas has resulted in the construction of inaccurate and misleading cognitive neuroscience models, and missed opportunities to explore the brain bases of this highly complex condition. However, if future research addresses this problem of male bias and associated implications for research programmes, then a deeper understanding of autism as a whole, as well as in previously overlooked females, will start to emerge.

## Data Availability

Not applicable.
